# A Simple and Effective Ratiometric Fluorescent Probe for the Selective Detection of Cysteine and Homocysteine in Aqueous Media

**DOI:** 10.3390/molecules21081023

**Published:** 2016-08-05

**Authors:** Risong Na, Meiqing Zhu, Shisuo Fan, Zhen Wang, Xiangwei Wu, Jun Tang, Jia Liu, Yi Wang, Rimao Hua

**Affiliations:** 1 Department of Science of Pesticides, School of Resources and Environment, Anhui Agricultural University, No. 130 Changjiang West Road, Hefei 230036, China; risongna@163.com (R.N.); zhumeiqing@ahau.edu.cn (M.Z.); wxw@ahau.edu.cn (X.W.); tangjun@ahau.edu.cn (J.T.); ljia198346@163.com (J.L.); 2 Collaborative Innovation Center of Henan Grain Crops, National Key Laboratory of Wheat and Maize Crop Science, College of Plant Protection, Henan Agricultural University, Wenhua Road No. 95, Zhengzhou 450002, China; 3 Department of Environmental Engineering, School of Resources and Environment, Anhui Agricultural University, No. 130 Changjiang West Road, Hefei 230036, China; fanshisuo@ahau.edu.cn (S.F.); zwang@ahau.edu.cn (Z.W.)

**Keywords:** fluorescent probe, cysteine and homocysteine, detection

## Abstract

Biothiols such as cysteine (Cys) and homocysteine (Hcy) are essential biomolecules participating in molecular and physiological processes in an organism. However, their selective detection remains challenging. In this study, ethyl 2-(3-formyl-4-hydroxyphenyl)-4-methylthiazole-5-carboxylate (**NL**) was synthesized as a ratiometric fluorescent probe for the rapid and selective detection of Cys and Hcy over glutathione (GSH) and other amino acids. The fluorescence intensity of the probe in the presence of Cys/Hcy increased about 3-fold at a concentration of 20 equiv. of the probe, compared with that in the absence of these chemicals in aqueous media. The limits of detection of the fluorescent assay were 0.911 μM and 0.828 μM of Cys and Hcy, respectively. ^1^H-NMR and MS analyses indicated that an excited-state intramolecular proton transfer is the mechanism of fluorescence sensing. This ratiometric probe is structurally simple and highly selective. The results suggest that it has useful applications in analytical chemistry and diagnostics.

## 1. Introduction

Biothiols such as cysteine (Cys), homocysteine (Hcy), and glutathione (GSH) are essential biomolecules and participate in molecular and physiological processes in living systems. Recently, they have received much research attention [1,2]. Cys is an important amino acid that not only plays a pivotal role in many physiological processes including protein synthesis, detoxification, and metabolism but is also very closely related to many serious diseases such as slow growth in children, liver damage, muscle and fat loss, skin lesions, and weakness [3,4,5]. Hcy has been proposed to be closely associated with the risk of Alzheimer’s disease [6], inflammatory bowel disease, and osteoporosis [7]. Therefore, the detection of Cys and Hcy is very important for potential disease diagnosis.

Various detection techniques such as fluorescence assays, high-performance liquid chromatography, capillary electrophoresis, immunoassays, and colorimetry [8,9] have been developed. Fluorescence detection is particularly attractive because of its high selectivity, high sensitivity, low cost, and its great potential for bioimaging with fluorescent probes [4,10,11,12,13,14,15,16,17,18,19,20]. Distinguishing Cys and Hcy from other biothiols, particularly GSH, remains a challenge because of their similar structures and reactivity [21,22]. Furthermore, the intracellular concentration of Cys (30–200 µM) is more than 10 times lower than that of GSH (1–10 mM), which makes the detection of Cys more difficult [23,24,25]. Many fluorescent probes have been developed for the detection of Cys/Hcy [4,26,27,28,29,30], however, most of these Cys/Hcy probes were based on fluorescence measurements at a single wavelength, which may be limited by many factors such as the instrument detection efficiency and environmental conditions. In contrast, ratiometric fluorescent probes allow the measurement of emission intensities at two different wavelengths, which provides a built-in correction of environmental effects and can also increase the dynamic range of the fluorescence measurement. Therefore, there is a need for ratiometric fluorescent probes for selective detection of Cys/Hcy to be developed.

**Figure 1:**
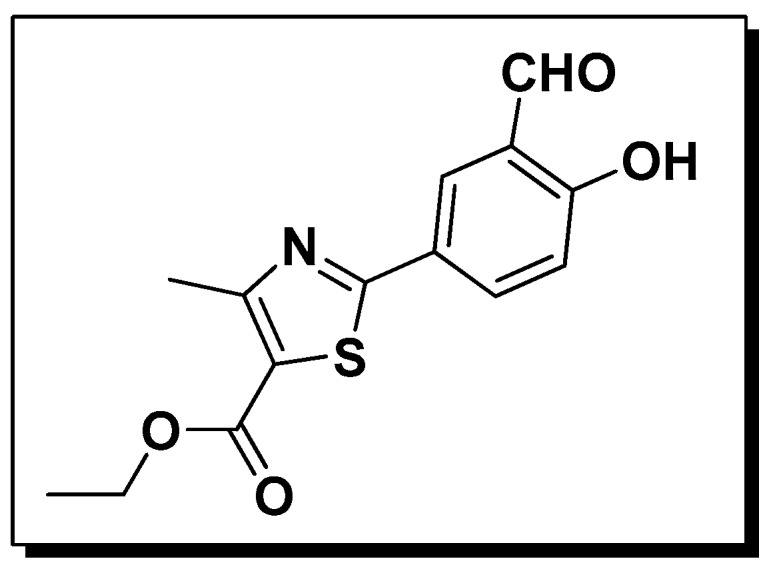
Structure of the fluorescent probe **NL**.

In the present study, we synthesized ethyl 2-(3-formyl-4-hydroxyphenyl)-4-methylthiazole-5-carboxylate (**NL**) (Figure 1) that can readily discriminate Cys/Hcy from other biothiols in approximately 2.5 h of detection. This probe undergoes an excited-state intramolecular proton transfer (ESIPT) process, which is confirmed by ^1^H-NMR and MS spectra. The **NL** probe is easy to synthesize (Supplementary Materials), versatile, and further structural modifications can improve its properties for biothiol detection.

## 2. Results and Discussion

**NL** was characterized and optimized as a fluorescent probe via studies of its UV-Vis absorption, fluorescence selectivity, reaction time of biothiols, quantification of Cys/Hcy, effect of pH on detection of Cys, as well as its detection mechanisms.

### 2.1. UV-Vis Absorption and Fluorescence Selectivity Studies of the **NL** Probe

**Scheme 1:**
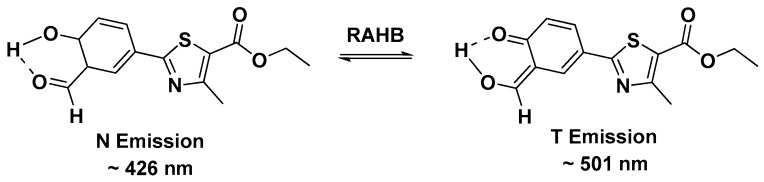
Probable isomers of **NL**.

First, the effect of dimethylsulfoxide (DMSO) content on the fluorescence intensity of the free **NL** probe and the behavior of the probe towards Cys were investigated. The fluorescence intensity of **NL** increased with increasing content of DMSO (Figure S3). In the presence of a polar aprotic solvent, the proton of the hydroxyl group is most likely strongly bonded with the nearest carbonyl group (Scheme 1). Buffer solutions were optimized based on the fluorescence signal-to-background ratio of **NL** with or without Cys in aqueous DMSO. The optimized solution was DMSO in 4-(2-hydroxyethyl)-1-piperazineethanesulfonic acid (HEPES) buffer (8:2, *v*/*v*, pH 7.4) and was used in the subsequent experiments.

**Figure 2:**
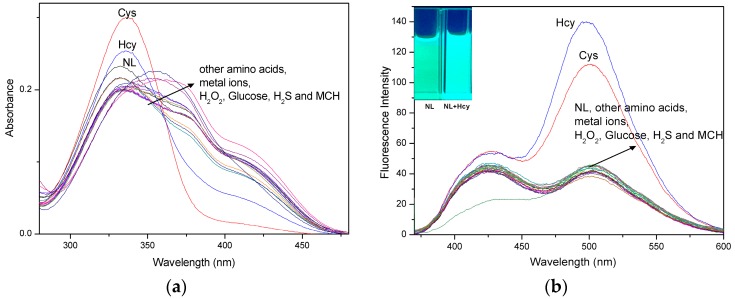
(**a**) UV-vis absorption spectra of the **NL** probe (10 µM) with various analytes (200 µM) in HEPES buffer solution (DMSO/HEPES = 8:2, pH 7.4); (**b**) Fluorescence spectra of **NL** (10 µM) with various analytes (200 µM) in HEPES buffer solution (DMSO/HEPES = 8:2, pH 7.4, λ_ex_ = 327 nm, slit: 2.0 nm/2.0 nm). Inset is a photograph of **NL** without and with Hcy upon excitation at 365 nm.

The selectivity of **NL** for Cys/Hcy measurements was evaluated with the DMSO/HEPES solution containing each of the 20 interference chemicals, in addition to the analytes of Cys and Hcy. The interference chemicals were GSH, arginine, aspartic acid, glutamic acid, glycine, histidine, lysine, proline, threonine, tryptophan, tyrosine, K^+^, Ca^2+^, Na^+^, Mg^2+^, Zn^2+^, H_2_O_2_, H_2_S, HSCH_2_CH_2_OH (MCH), and glucose, each at a concentration of 20-fold compared to the **NL** probe. Cys/Hcy increased the absorbance of the solutions at 327 nm, while the other chemicals did not increase the absorbance of the solutions (Figure 2a). In addition, fluorescence intensity at λ_em_ 501 nm was markedly enhanced in the presence of Cys/Hcy (Figure 2b). The other analytes caused negligible effects on the fluorescence intensity of **NL**. In particular, GSH did not affect the UV-Vis absorbance and fluorescence intensity of the probe (Figure 2a,b, Figures S4 and S5). The presence of a wide range of interferences had little effect on the detection of Cys/Hcy. Therefore, **NL** possesses good selectivity toward Cys/Hcy in the presence of other amino acids and metal ions as well as GSH.

### 2.2. Reaction Time of Biothiols

To investigate the reaction time of **NL** with biothiols, the fluorescence intensity at λ_em_ 501 nm was recorded at varying periods of time after additon of Cys, Hcy, and GSH to the **NL** solutions (Figure S6). The reaction of **NL** with Cys or Hcy was nearly completed within 2.5 h at ambient temperature. These reactions are relatively fast compared with those reported in the literature [31,32]. In contrast, there was no obvious change in the fluorescence intensity of the solution containing GSH during the measurement time. Some reported probes can detect biothiols rapidly but with large amounts of biothiols, such as 30 equiv. or even more than 1000 equiv. of the probe [33,34,35,36]. In this study, we used 20 equiv. to achieve an accurate measurement of the biothiols.

### 2.3. Quantification of Cys/Hcy

**Figure 3:**
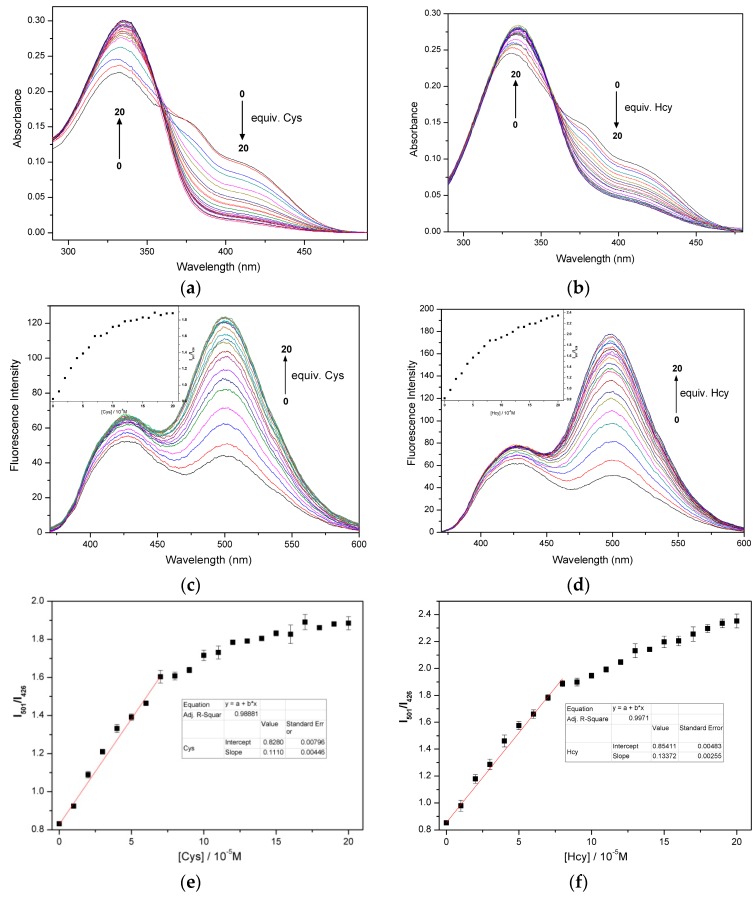
(**a**,**b**) UV-vis absorption spectra of **NL** (10 µM) with Cys/Hcy (0, 1, 2, 3, 4, 5, 6, 7, 8, 9, 10, 11, 12, 13, 14, 15, 16, 17, 18, 19 and 20 equiv.) in HEPES buffer solution (DMSO/HEPES = 8:2, pH 7.4); (**c**,**d**) Fluorescence spectra of **NL** (10 µM) with Cys/Hcy (0, 1, 2, 3, 4, 5, 6, 7, 8, 9, 10, 11, 12, 13, 14, 15, 16, 17, 18, 19 and 20 equiv.) in HEPES buffer solution (DMSO/HEPES = 8:2, pH 7.4, λ_ex_= 327 nm, slit: 2.0 nm/2.0 nm). Inset is the corresponding ratiometric response (I_501_/I_426_) of **NL** to the addition of Cys/Hcy; (**e**,**f**) Plot of the ratiometric response (I_501_/I_426_) of **NL** (10 µM) against Cys/Hcy equiv.; data are mean ± standard error (bars) (*n* = 3).

Figure 3a,b show the change in the UV-Vis absorbance of the solutions containing the **NL** probe with increasing content of Cys/Hcy. The absorbance intensity at 327 nm increased, but the intensity at 426 nm decreased with increasing Cys/Hcy concentration. Figure 3c,d reveal that the fluorescence intensity of the probe solution increased at λ_em_ 501 nm with increasing content of Cys/Hcy. When Cys/Hcy was at 20 equiv. of **NL**, the fluorescence intensity and the ratio of the absorption intensities at 501 and 426 nm (I_501_/I_426_) were almost at their maximum values, which is a superior performance in comparison with some other probes [33,37]. In addition, we found a linear correlation (R^2^ = 0.9888) between the fluorescence intensity and Cys concentration from 1–7 µM. The limit of detection (LOD) of Cys was 0.911 µM (Figure 3e), which was calculated by S/N = 3. A similar response of the **NL** probe to Hcy was also observed, as shown in Figure 3f. In this case, the LOD was 0.828 µM based on the linear relationship (R^2^ = 0.9971) between the fluorescence intensity and Hcy concentration in the range of 1–8 µM. In comparison with the previously reported probes [20,38,39,40], **NL** provides a much lower LOD of Cys/Hcy for qualitative analysis of Cys/Hcy despite the presence of GSH (Figure S7), making it attractive for use in human plasma samples [28].

### 2.4. Effect of pH on Detection of Cys

The effect of pH on the response of **NL** to Cys is illustrated in Figure S8. In the presence or absence of Cys, **NL** was stable under acidic conditions. With increasing pH from 2.0–8.0, the fluorescence intensity of the solution increased until it reached its maximum value at pH 8.0. Weak alkaline conditions possibly promoted the reaction between Cys and NL. With a further increase of the pH, the fluorescence intensity of the solution decreased until it was quenched, which might be caused by the hydrolysis of ester groups or the opening of the thiazolidine ring under strong alkaline conditions. Therefore, we chose pH 7.4 as a suitable pH for the detection experiments. Unlike the early and even recently reported probes in the literature that require use at pH ≥ 9.0 [28,41,42], **NL** can function in the physiological pH range.

### 2.5. Mechanism of **NL** in Detection of Cys/Hcy

**Scheme 2:**
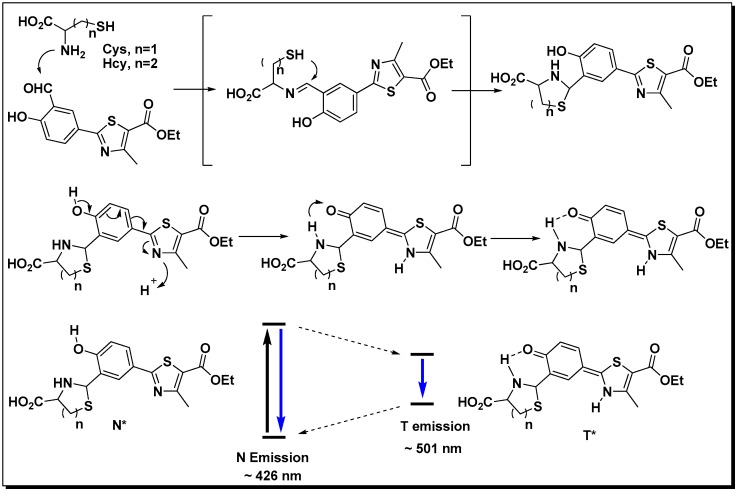
Proposed reaction mechanism of **NL** and Cys/Hcy.

To verify the detection mechanism between **NL** and Cys/Hcy as shown in Scheme 2, we investigated the ^1^H-NMR spectrum of **NL** upon the addition of Cys and compared it with that of the probe itself. Under the same conditions, Hcy also reacted similarly with **NL** and formed the six-membered thiazinane ring. After the reaction of **NL** with Cys/Hcy in DMSO-*d*_6_ for 4 h, the reaction mixture was analyzed with NMR, which indicated that the aldehyde proton at around δ 10.3 ppm had disappeared, and that the thiazolidine methane proton at around δ 5.5 ppm had newly appeared (Figure S2). Furthermore, UPLC-MS/MS were carried out to confirm that the thiazolidine ring was formed by the reaction of Cys/Hcy with the aldehyde group of **NL**. The mass spectrum (ESI MS) of the **NL** probe shows a peak at *m*/*z* 292.09 (**NL** + H)^+^. The peak of the product of **NL** + Cys is 394.93, which also proves a single mononuclear addition of Cys to **NL**. A peak at *m*/*z* 408.92 corresponding to the product of **NL** + Hcy was also observed (Figure S2).

**NL** at λ_ex_ 327 nm produced two emission peaks at ~426 nm and ~501 nm. Adding Cys/Hcy to **NL** enhanced the ESIPT process to shift the emission signal to a longer wavelength. As a result, the fluorescence intensity at λ_em_ 501 nm, which was attributed to the tautomer (T* emission) of the Cys/Hcy-products, was enhanced strongly, and the normal isomer (N* emission) of the products caused the fluorescence intensity at 426 nm to increase slowly. The two emission peaks can be used for the ratiometric fluorescent measurement to determine the presence of the analytes more accurately with the minimization of the background signal [43].

## 3. Experimental Section

### 3.1. General Methods and Materials

Commercial reagents were used as obtained from Sigma-Aldrich (St. Louis, MO, USA), Alfa Aesar (Ward Hill, MA, USA), and J&K (Beijing, China). Twice-distilled water was used throughout all the experiments. Nuclear magnetic resonance (NMR) spectra were recorded with a 600-MHz spectrometer (Agilent Technologies, Inc., Santa Clara, CA, USA) and mass spectrometry (MS) was performed with a UPLC/XevoTQ MS/MS spectrometer (Waters, Milford, MA, USA). All fluorescence measurements were carried out on a 970CRT fluorescence spectrophotometer (Inesa, Shanghai, China) equipped with a xenon lamp source. UV-Vis spectra were measured on a UV-1800 spectrophotometer (Shimadzu, Kyoto, Japan). Quartz cuvettes with a 1-cm path length and 3-mL volume were used for all measurements. All pH measurements were completed with a PHS-25 digital pH meter (Shanghai REX Instrument Factory, Shanghai, China).

### 3.2. Optical Studies of the **NL** Probe upon Addition of Various Analytes

A stock solution of the **NL** probe with a concentration of 12.5 µM was prepared in DMSO. Amino acids (Cys, Hcy, GSH, arginine, aspartic acid, glutamic acid, glycine, histidine, lysine, proline, threonine, tryptophan, and tyrosine), cations (K^+^, Ca^2+^, Na^+^, Mg^2+^ and Zn^2+^), H_2_S, and HSCH_2_CH_2_OH (MCH) were all dissolved in distilled water at a concentration of 5 mM for the absorption and fluorescence spectra analyses. The stock solutions of the analytes were diluted to various concentrations with HEPES buffer. Test solutions were prepared by adding 2400 µL of the **NL** stock solution (the final concentration of **NL** was 10 µM) and an appropriate aliquot of test analyte into a 3-mL volumetric cuvette. Each solution was diluted to 3 mL using a mixture of DMSO and HEPES buffer (8:2, *v*/*v*) at pH 7.4. The resulting solution was shaken well and incubated for 2.5 h at room temperature. Fluorescent spectra were then recorded using an excitation wavelength of 327 nm and scan speed of 450 nm·min^−1^.

## 4. Conclusions

In order to find a simple method to distinguish Cys/Hcy from other amino acids at physiological pH, an effective ratiometric fluorescent **NL** probe was developed. The fluorescence intensity of the probe was enhanced 3-fold upon addition of Cys/Hcy at a concentration of 20 equiv. of the probe, and its absorbance in buffer solution was also increased. **NL** can detect Cys at concentrations of 0.911 to 20 µM and Hcy at concentrations of 0.828 to 20 µM.

## Supplementary Materials

Supplementary materials can be accessed at: http://www.mdpi.com/1420-3049/21/8/1023/s1.
