# Supplementary material for: A Simple and Effective Ratiometric Fluorescent Probe for the Selective Detection of Cysteine and Homocysteine in Aqueous Media
Source: Molecules. 2016 Aug 5;21(8):1023. doi: 10.3390/molecules21081023 (PMC6274303; doi:10.3390/molecules21081023)
Supplement: Supplementary file 1 [file molecules-21-01023-s001.pdf]

Supplementary Materials: A Simple and Effective Ratiometric Fluorescent Probe for the Selective Detection of Cysteine and Homocysteine in Aqueous Media

Risong Na, Meiqing Zhu, Shisuo Fan, Zhen Wang, Xiangwei Wu, Jun Tang, Jia Liu, Yi Wang and Rimao Hua

1. Synthesis of the NL probe

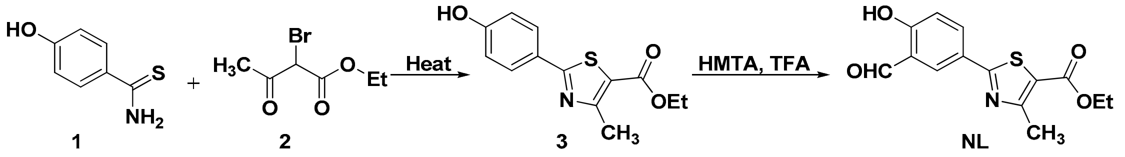

Scheme S1. Synthesis routes for the **NL** fluorescent probe.

4-Hydroxybenzothioamide **1** (20 mmol), 2-bromo-3-oxobutyric acid ethyl ester **2** (24 mmol) and absolute ethanol (30 mL) were added to a round-bottomed flask. The mixture was refluxed for 3 h. After cooling to room temperature, the green solid was filtered off, and washed with ethanol. The crude product recrystallized from ethanol to give compound **3** in 91% yield. Yellow solid, m.p. 187–190 °C.

Compound **3** (24 mmol) and HMTA (1 mL) and trifluoroacetic acid (30 mL) were added to a round-bottomed flask. The mixture was refluxed for 20 h. After completion of the reaction, the solvent was removed by distillation. The residue was poured into ice water and stirred vigorously, the solid product was filtered and washed with water, then dried in a vacuum to give compound **NL**, recrystallized in toluene in 80% yield. Yellow solid; m.p. 97.0–99.0 °C. ^{1}H-NMR (DMSO-d_6): δ 10.32 (s, 1H), 8.22 (d, *J* = 2.4 Hz, 1H), 8.09 (dd, *J* = 8.7, 2.5 Hz, 1H), 7.13 (d, *J* = 8.7 Hz, 1H), 4.29 (q, *J* = 7.0 Hz, 2H), 2.67 (s, 3H), 1.30 (t, *J* = 7.1 Hz, 3H), ^{13}C-NMR (DMSO-d_6): δ 190.62, 168.51, 163.52, 161.80, 160.71, 134.50, 127.17, 124.04, 123.05, 120.83, 118.86, 61.59, 17.65, 14.58. HRMS (ESI) calcd for $C_{14}H_{12}NO_4S^-$ m/z $[M-H]^-$ 290.0493, found 290.0487.

2. Supplementary Figures

^{1}H-NMR of NL

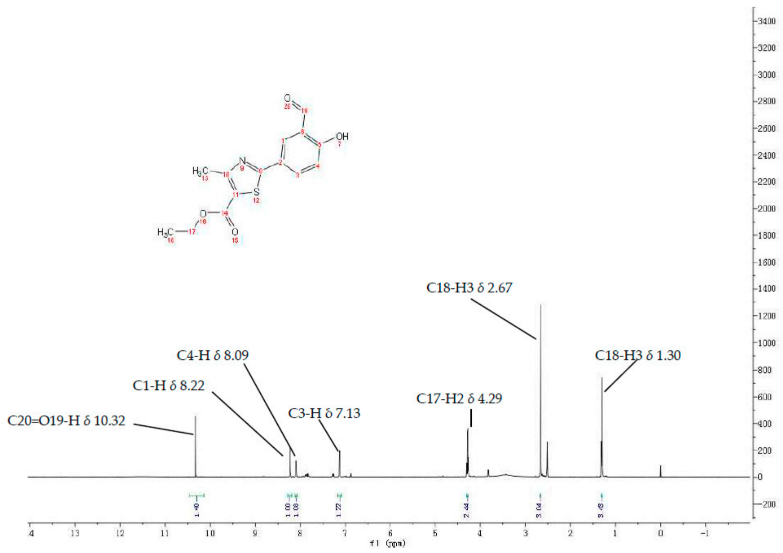

^{13}C-NMR of NL

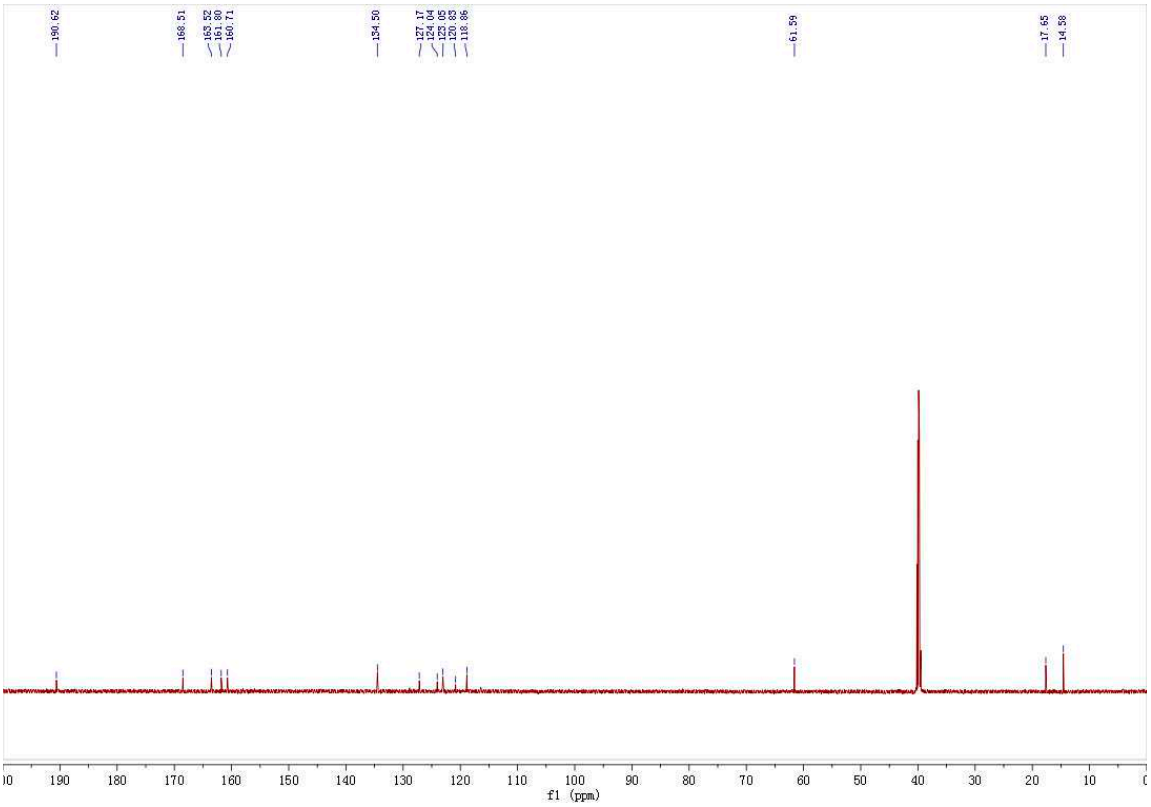

^{1}H-NMR of NL + Hcy

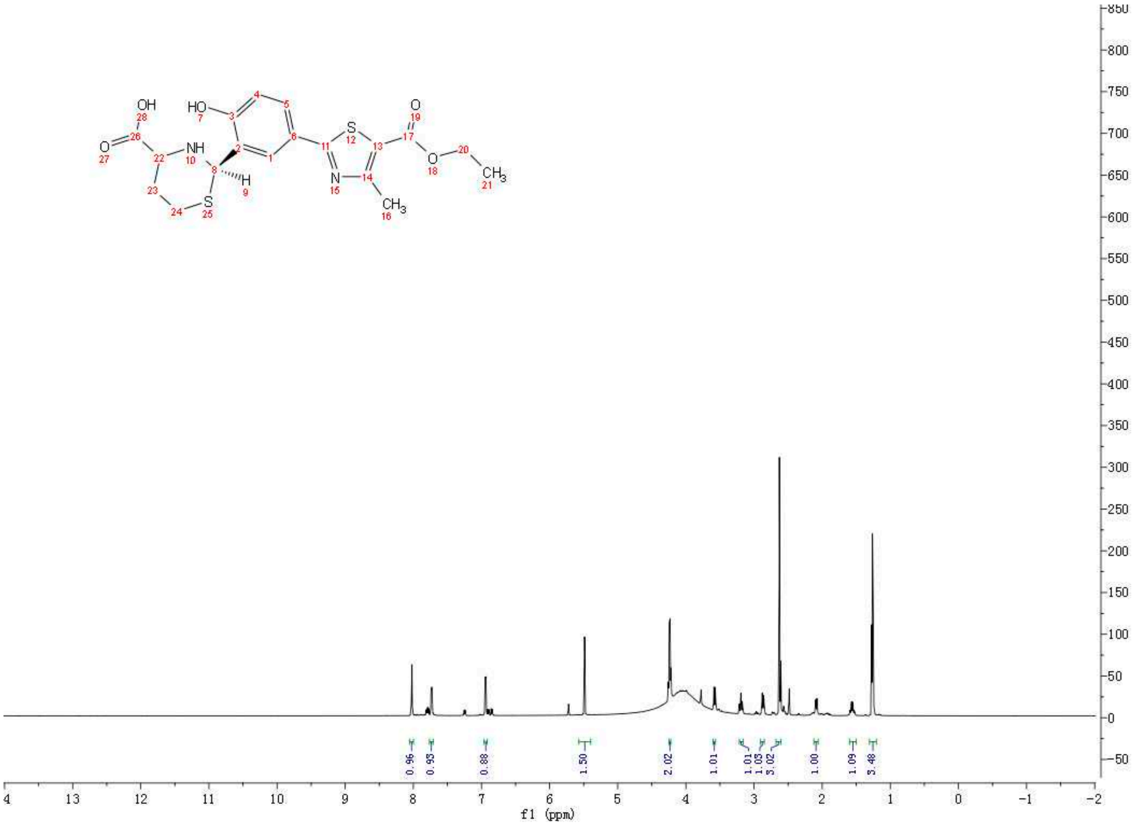

Comparison of ^{1}H-NMR of NL and NL + Hcy

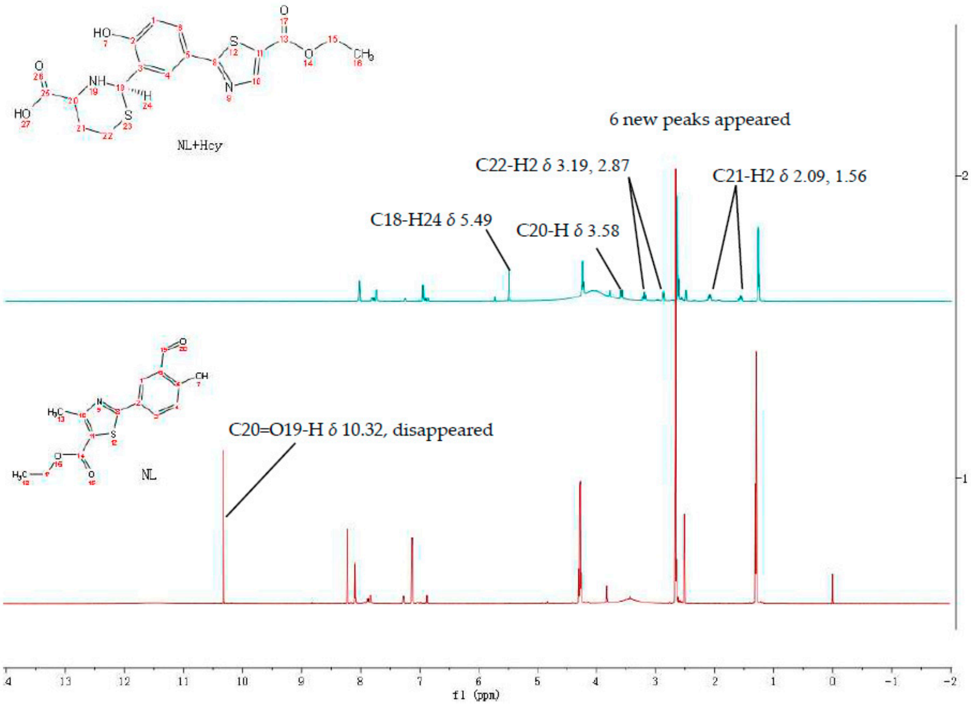

Figure S1. NMR Spectra for **NL** and **NL** + Hcy.

UPLC-MS chromatogram and spectrum of NL

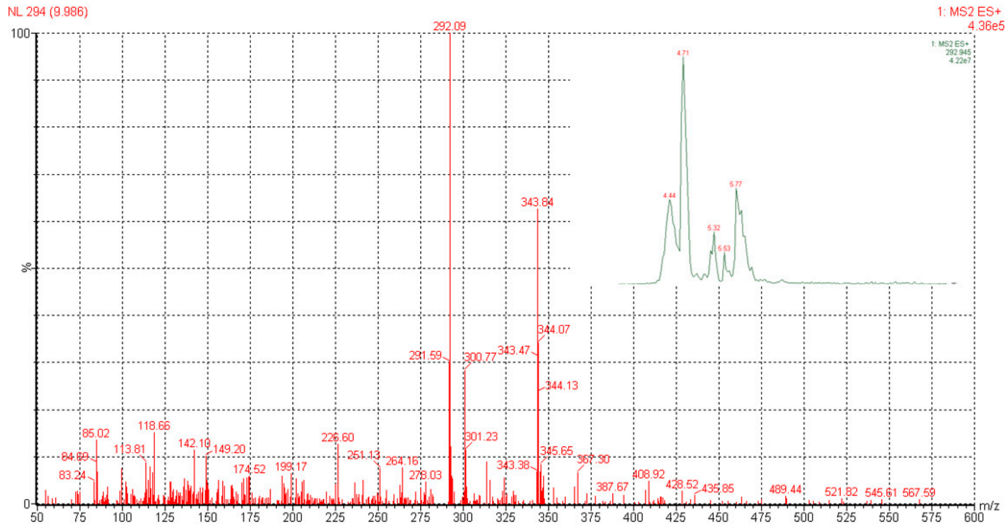

UPLC-MS chromatogram and spectrum of NL + Cys

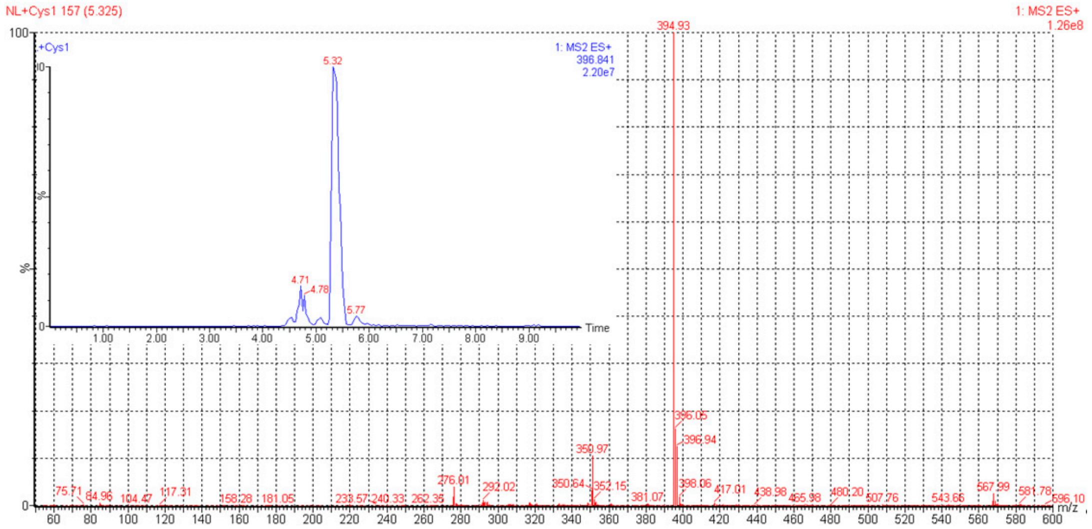

UPLC-MS chromatogram and spectrum of NL + Hcy

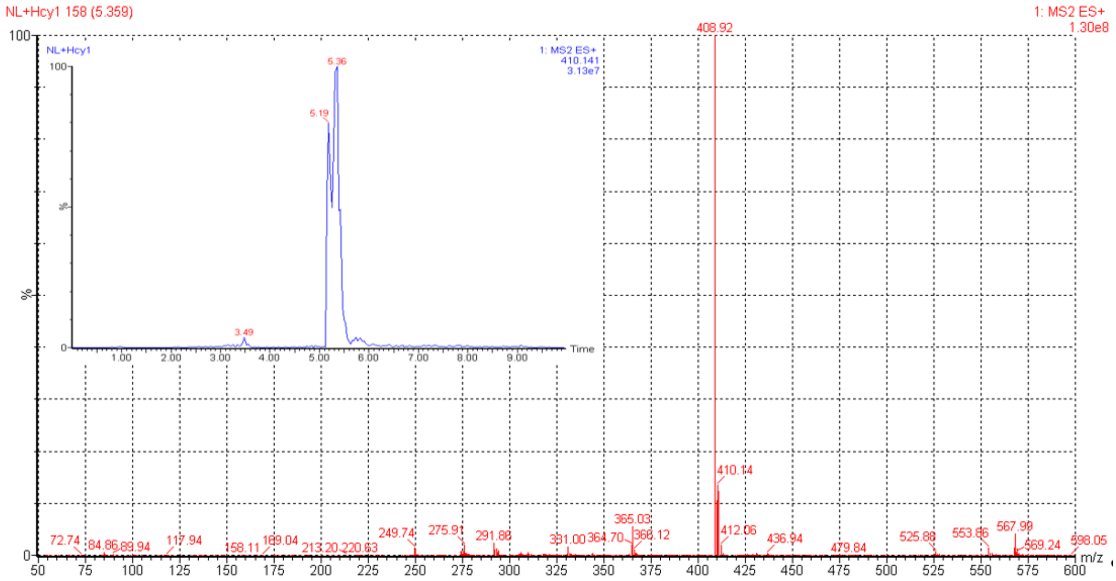

Figure S2. UPLC-MS chromatograms and spectra of **NL**, **NL** + Cys, and **NL** + Hcy.

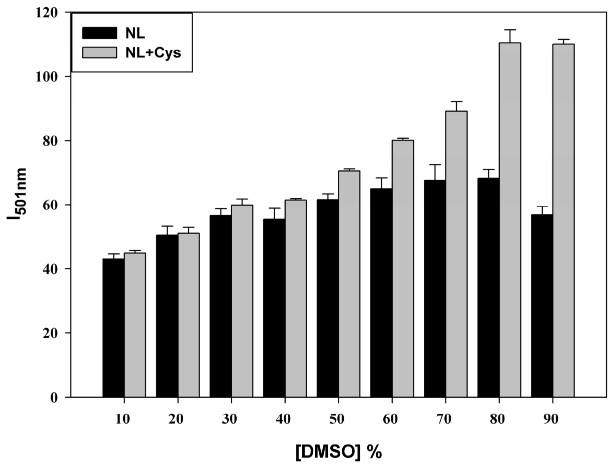

Figure S3. Fluorescence response of **NL** (1 × 10^{-5} M) without (black) and with (gray) Cys (2 × 10^{-4} M) in HEPES buffer solution with different ratios of DMSO and HEPES. (pH 7.4, λ_{ex} = 327 nm, slit: 2.0 nm/2.0 nm).

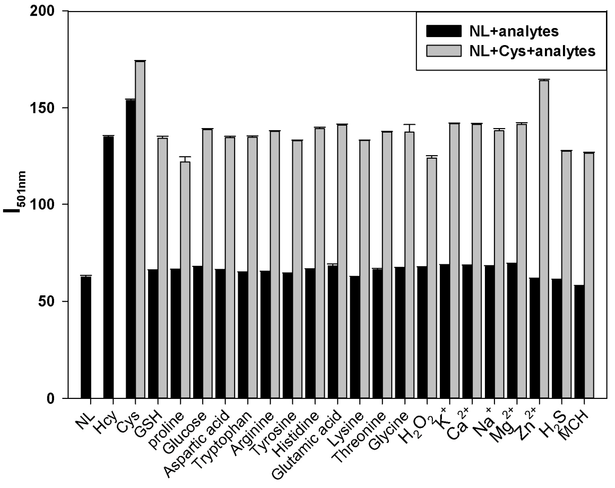

Figure S4. Fluorescence response of **NL** (1 × 10^{-5} M) toward Cys (2 × 10^{-4} M) without and with other analytes (2 × 10^{-4} M) in HEPES buffer solution (DMSO/HEPES = 8:2, pH 7.4) at 501 nm (λ_{ex} = 327 nm, slit: 2.0 nm/2.0 nm).

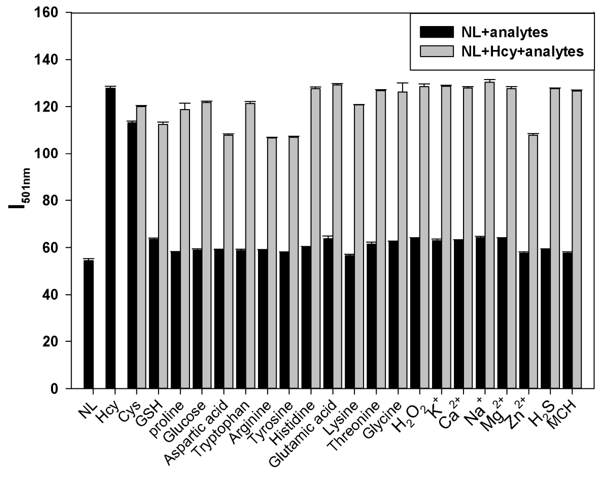

Figure S5. Fluorescence response of **NL** (1 × 10^{-5} M) toward Hcy (2 × 10^{-4} M) without and with other analytes (2 × 10^{-4} M) in HEPES buffer solution (DMSO/HEPES = 8:2, pH 7.4) at 501 nm (λ_{ex} = 327 nm, slit: 2.0 nm/2.0 nm).

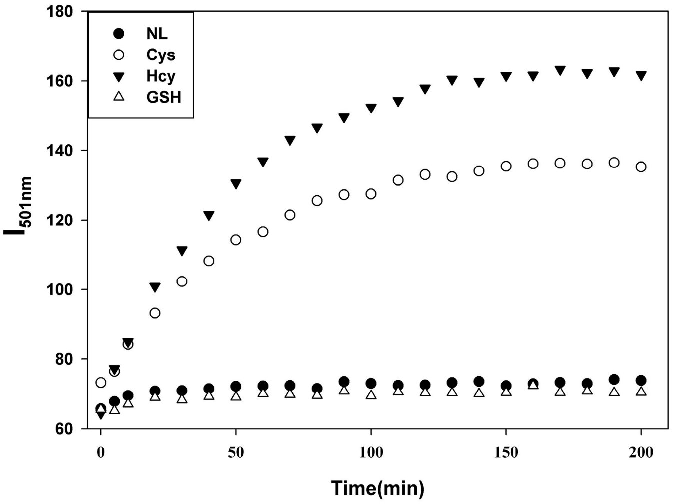

Figure S6. Time-dependent fluorescence intensity at 501 nm of **NL** (1×10^{-5} M) without and with Cys (2×10^{-4} M), Hcy (2×10^{-4} M), and GSH (2×10^{-4} M) in HEPES buffer solution (DMSO/HEPES = 8:2, pH 7.4, λ_{ex} = 327 nm, slit: 2.0 nm/2.0 nm).

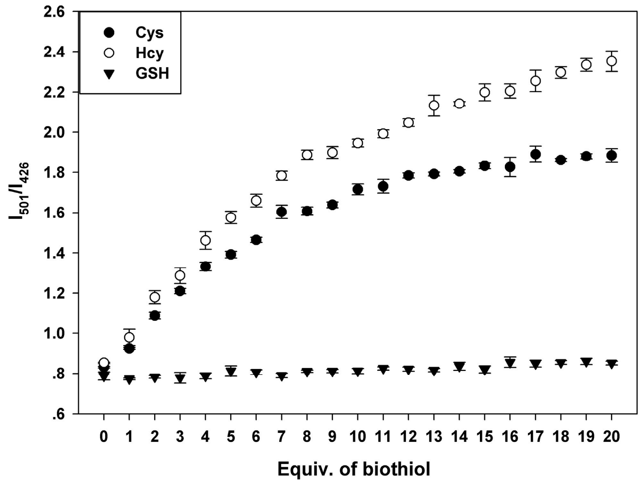

Figure S7. Fluorescence intensity ratio at 426 nm and 501 nm with various concentrations of Cys, Hcy, and GSH. **NL** (1×10^{-5} M) in HEPES buffer solution (DMSO/HEPES = 8:2, pH 7.4, λ_{ex} = 327 nm, slit: 2.0 nm/2.0 nm). Data are means ± SE (bars) (n = 3).

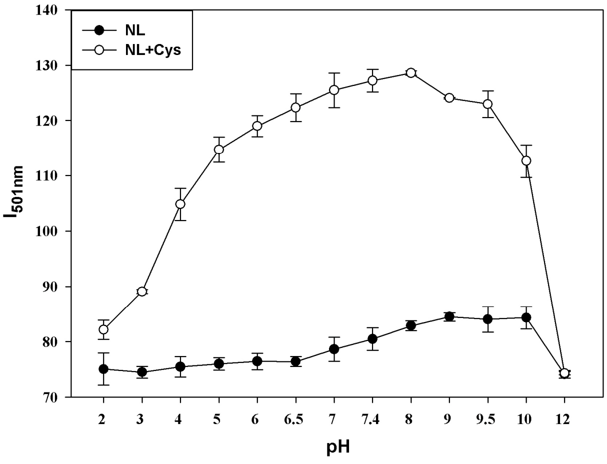

Figure S8. Fluorescence response of **NL** (1×10^{-5} M) without or with Cys (2×10^{-4} M) in HEPES buffer solution (DMSO/HEPES = 8:2, λ_{ex} = 327 nm, slit: 2.0 nm/2.0 nm) at different pH.
